# Drugs and Medicines of North America

**Published:** 1887-09

**Authors:** 


### BOOK NOTICES AND REVIEWS.

Drugs and Medicines of North America. A quarterly. J. U. & C. G. Lloyd, editors and publishers. Cincinnati, 1887.

No. 4 of volume II of this grand work has been issued. It contains the botany of *Lobelia syphilitica*, also gives the botany and pharmacy of Scrophularia or Figwort, Lindera, Benzoin, or Spicebush, etc.

As we have repeatedly stated in noticing previous issues of this wonderful work, it gives the botany, origin of name, chemistry, pharmacy, and medical uses of the various plants treated. Then comes the medical uses of the plant according to ALL schools of medicine, and finally is given the bibliography.

Thus the work done is thorough, the cuts and plates are excellent, though without coloring.

Considering the scope of the work, its thoroughness, and its freedom from bias in giving the testimony of men of all schools of medicine, it is invaluable to every practitioner. Its price, one dollar per year, puts it within the reach of all. We see no good reason why every one of the ten thousand homœopaths in the United States should not be a subscriber.

We hope to hear of every one of our own readers taking it.

